# Appearance of *vanD*-positive *Enterococcus faecium* in a tertiary hospital in the Netherlands: prevalence of *vanC* and *vanD* in hospitalized patients

**DOI:** 10.1038/s41598-019-42824-4

**Published:** 2019-05-06

**Authors:** Jacky Flipse, Christian J. H. von Wintersdorff, Julius M. van Niekerk, Casper Jamin, Frank H. van Tiel, Henrik Hasman, Lieke B. van Alphen

**Affiliations:** 1 Department of Medical Microbiology, Care and Public Health Research Institute (CAPHRI), Maastricht University Medical Center+, Maastricht, The Netherlands; 2 Present Address: Laboratory for Medical Microbiology and Infectious Diseases, Isala Clinics, Zwolle, The Netherlands; 3 Department of Bacteria, Parasites and Fungi, Statens Serum Institut, Copenhagen, Denmark

**Keywords:** Antimicrobial resistance, Clinical microbiology, Public health

## Abstract

Vancomycin-resistant enterococci (VRE) can rapidly spread through hospitals. Therefore, our hospital employs a screening program whereby rectal swabs are screened for the presence of *vanA* and *vanB*, and only PCR-positive broths are cultured on VRE selection agar. Early November 2016, a clinical *vanA-*/*vanB*-negative VRE isolate was detected in a *vanA/vanB*-screening-negative patient, giving the possibility that an undetected VRE might be spreading within our hospital. Whole-genome-sequencing of the isolate showed that resistance was *vanD*-mediated and core genome multilocus sequence typing showed it was a rare type: ST17/CT154. To determine the prevalence of *vanA/B/C/D*-carrying enterococci, we designed a real-time PCR for *vanC1/2/3* and *vanD* and screened rectal swabs from 360 patients. *vanD* was found in 27.8% of the patients, yet culture demonstrated only *E*. *faecium* from *vanA*-positive broths and *E*. *gallinarum* from *vanC1*-positive broths. No *vanD*-positive VRE were found, limiting the possibility of nosocomial spread of this VRE. Moreover, the high prevalence of non-VRE *vanD* in rectal swabs makes it unfeasible to include the *vanD* PCR in our VRE screening. However, having validated the *vanC1/2/3* and *vanD* PCRs allows us to rapidly check future *vanA/B*-negative VRE for the presence of *vanC* and *vanD* genes.

## Introduction

Vancomycin-resistant enterococci (VRE) may be considered as an important healthcare-associated pathogen, especially in immunocompromised patients^1,2^. Acquisition of vancomycin resistance is mediated by several *van* gene clusters^3^. The most commonly encountered vancomycin resistance genes are *vanA* and *vanB*, which confer either high level resistance to vancomycin (MIC > 128 mg/L) or more moderate levels (16 to 64 mg/L), respectively^3,4^. Moreover, the localization of these *vanA-* and *vanB-containing* pathogenicity islands on mobile genetic elements allow this type of resistance to spread clonally and laterally. Therefore, these resistance genes are highly transferable between enterococci and thus pose a risk for nosocomial spread^5^. Consequently, several public health institutes, including our hospital, now screen for the carriage of *vanA*- and *vanB*-positive VRE in hospitalized patients^6^.

In contrast to *vanA* and *vanB*, *vanC* and *vanD* in VRE are chromosomally associated, i.e. much less transferable^7^. *vanC* generally presents with low-level resistance against vancomycin (MIC 2–32 mg/L) and teicoplanin (≤1 mg/L)^3^. Moreover, VRE containing *vanC* are typically *E*. *gallinarum* or *E*. *casseliflavus*/*E*. *flavescens*, and associated with low risk of mortality and rarely cause nosocomial outbreaks^8–11^. Hence, *vanC*-positive patients typically are not isolated, contrary to patients with *vanA*- or *vanB*-positive VRE^9^. The *vanD* gene, generally, confers moderate-level resistance against vancomycin (MIC 64–128 mg/L) and teicoplanin (MIC 4–64 mg/L) and is constitutively expressed^3,12^. Until recently, *vanD* was mostly found in anaerobic commensals of the human gut microbiome^13^ and was seldom found in enterococci. In Europe, individual cases with *vanD*-positive enterococci have been reported in patients from France (2005)^14^ and Sweden (2007, 2016)^15,16^. Yet, recently six clinical *vanD*-positive *E*. *faecium* isolates were reported from the Netherlands^3,17^. These isolates presented with MIC values of 8–256 mg/L (vancomycin) and <0.5–4 mg/L (teicoplanin)^17^. Overall, *vanD*-positive enterococci are relatively unknown and, as far as we know, are not included in surveillance screenings. Thus, VRE surveillance programs might miss the presence of *vanD*-positive enterococci within a hospital.

Screening for VRE carriage can be useful to identify unrecognized cases to prevent nosocomial transmission of VRE and reduce the subsequent risk of VRE infection^18^. Therefore, the Maastricht University Medical Centre (MUMC+) adopted a monthly point prevalence screening of all admitted patients in all wards of the hospital. As part of the screening, a rectal swab is taken from a patient, cultured in TSB broth, and subsequently screened with *vanA/vanB* PCR. PCR-positive broths are then inoculated on VRE selection agar to determine whether a vancomycin-resistant *Enterococcus faecium* or *Enterococcus faecalis* is present. Parallel to the screening described above, high-risk wards (e.g. Intensive Care, gastroenterology) also submit rectal swabs to screen for ESBL and VRE by culture on selection agars without prior *vanA/vanB* PCR.

The first VRE isolate of each patient is typed by conventional multilocus sequence typing (MLST). Epidemiological data and typing results are combined to assess whether nosocomial transmission is likely. In case of expected nosocomial transmission, patients are isolated, isolates are whole-genome-sequenced, specific cleaning procedures are implemented and screening of the ward is intensified.

## Case Description

**Figure 1:**
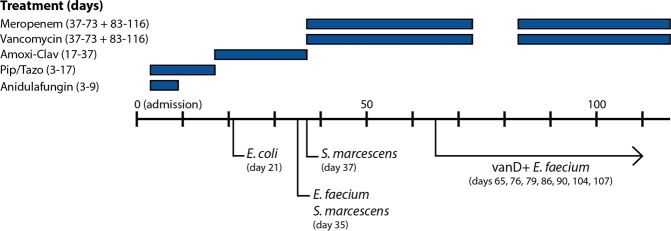
The time line of the patient, who initially presented with acute severe necrotizing pancreatitis on day 0 (admission). Over time, various microorganisms were isolated from clinical samples: *E*. *coli* on day 21 from fine needle aspirate (sensitive to all β-lactam antibiotics), *Serratia marcescens* (pip/tazo resistant) and *Enterococcus faecium* (amoxicillin resistant, vancomycin susceptible) on day 35 from transgastric drainage fluid. *Serratia marcescens* was also isolated from blood cultures on day 37. From day 65 on, a vancomycin resistant *E*. *faecium* was also isolated from rectal swabs. Earlier rectal swabs did not produce vancomycin resistant enterococci. Abbreviations: Amoxi-Clav (Amoxicillin-clavulanic acid), Pip/Tazo (piperacillin-tazobactam).

Within our hospital, a 59 year-old male presented with acute, severe necrotizing pancreatitis, following heavy alcohol consumption. Computed tomography scan revealed a necrotizing pancreatitis and intra-abdominal air, without a known cause. Although initially clinically stable, the patient was transferred to the Intensive Care Unit due to respiratory failure and progressive pancreatic necrosis. Over the course of four months, various isolates were cultured from aspirations and drainages (Fig. 1). The isolation of *Serratia marcescens* and *E*. *faecium* from transgastric fluid and blood cultures prompted an antibiotic switch to meropenem and vancomycin.

Moreover, the patient was routinely screened twice-weekly for carriage of VRE, by directly inoculating the rectal swabs on VRE selection agar without PCR-based selection. During the first one and half month, thirteen swabs were taken and none yielded VRE. Yet, on day 65 of admission, a vancomycin-resistant *E*. *faecium* was isolated which had MIC values of 16 mg/L for vancomycin, 1 mg/L for teicoplanin, and 2 mg/L for linezolid (*vanD-65*). The isolate was analyzed by PCR and proved to be negative for *vanA* and *vanB*. Hereafter, more VRE were isolated from rectal swabs of this patient (Fig. 1). The last known VRE isolate was found on day 107 of admission (*vanD-107*), which had MIC values of 24 mg/L for vancomycin, 1 mg/L for teicoplanin, and ≥8 mg/L for linezolid (Table 1). On the 116^th^ day of admission, antibiotic therapy was discontinued. In total, the patient had received meropenem and vancomycin for 73 days. Follow-up testing was performed at one year after discharge and the patient was asked to submit ten rectal swabs taken at least 12 h apart (i.e. spanning ≥5 days). None of the swabs yielded VRE.

**Table 1:** An overview of various characteristics of the *vanD*-positive *Enterococcus faecium* described in this report and the six previously described Dutch *vanD*-positive *E*. *faecium* isolates.

*Reference*	*Isolate*	*Resistance*	*Hospital day*	MLST type	*vanD* type	Patient	MIC values (mg/L)			
							Amoxicillin	Vancomycin	Teicoplanin	Linezolid
This report	VSE	ARE	35	Unknown	—	F	≥32	≤0.5	≤0.5	2.0
	*vanD-65*	VRE	65	17	*vanD4*	F	≥256	16	1.0	2.0
	*vanD-76*	VRE	76	17	*vanD4*	F	≥256	32	1.0	2.0
	*vanD-79*	VRE	79	17	*vanD4*	F	≥256	16	1.0	2.0
	*vanD-86*	VRE	86	17	*vanD4*	F	≥256	24	1.0	2.0
	*vanD-90*	VRE	90	17	*vanD4*	F	≥256	16	1.0	4.0
	*vanD-104*	VRE	104	17	*vanD4*	F	≥256	24	1.0	4.0
	*vanD-107*	VRE	107	17	*vanD4*	F	≥256	24	1.0	≥8.0
Top *et al*.^17^	E7962	VRE		897	*vanD4*	A	>256	16	<1	N.R.
	E8043	VRE		203	*vanD4*	B	>256	16	<1	N.R.
	E9242	VRE		262	*vanD2*	C	>256	256	4	N.R.
	E9352	ARE		117	—	C	>256	<1	<1	N.R.
	E9353	ARE		117	—	D	>256	<1	<1	N.R.
	E8429	VRE		1180	*vanD2*	D	>256	256	2	N.R.
	E9354	VRE		1281	*vanD2*	D	>256	256	2	N.R.
	E9641	VRE		1296	*vanD4*	E	>256	8	<0.5	N.R.

Shown are MLST types, *vanD* subtypes and the MIC values for amoxicillin, vancomycin, teicoplanin, linezolid. MIC values for this report were determined by E-test for isolates 65–107. Only VITEK II data is known for the vancomycin-susceptible isolate of hospital day 35. The VRE isolates of day 65 through day 107 were re-evaluated by E-test (Shown is median of 3 independent measurements). VSE: vancomycin susceptible enterococcus, VRE: vancomycin resistant enterococcus, ARE: ampicillin resistant enterococcus. N.D.: not reported.

## Results

### Vancomycin-resistant isolate

The vancomycin-resistant *E*. *faecium* isolated on hospital day 65 (*vanD-65*) was negative for *vanA* and *vanB* by PCR. The species identity was confirmed by biochemical properties (resistant for furazolidone, sensistive for both mupirocine and tellurite), MALDI-TOF and 16S sequencing. To determine the origin of the vancomycin resistance, whole genome sequencing (WGS) was performed at the Statens Serum Institut (SSI) in Denmark.

Assembly resulted in 246 contigs, genome size 3102488 bp. The resulting genome was analyzed for resistance genes using ResFinder 2.1^19^, which identified a full *vanD* gene cluster, located on position 198734..104387 of contig 1 (contig length = 192.330 bp; Hit length 5654 bp) with a 99,94% identity to the *vanD* cluster in the species *Enterococcus raffinosus* (Strain GV5; GenBank: AB242319.1). Additional resistance genes were identified; three coding for aminoglycoside resistance and two for macrolide resistance, respectively (Supplement A1).

Interestingly, both the left and right flank of contig 1 showed >99% identity to fully sequenced *E*. *faecium* chromosomes such as Aus0085 (Genbank: CP006620.1), while a large internal fragment of contig 1 (approximately 168.900 bp containing the *vanD* gene cluster) showed very little similarity to any sequences at NCBI except from some DNA fragments ranging between <100 bp and 21892 bp with 88–97% identity to a *Blautia* (previously *Clostridium*) *coccoides* genome (Strain YL58; GenBank NZ CP022713) and the *vanD* gene cluster of *E*. *raffinosus* mentioned above.

Subsequent MLST and cgMLST analysis at the SSI showed the isolate to be MLST type ST17, clonal type CT154 (ST17/CT154), a strain rarely found in the cgMLST database. The cgMLST database contained six other ST17/CT154 VRE. However, none of these contained a *vanD* gene cluster, and contained various other additional resistance genes (Supplement A1).

**Figure 2:**
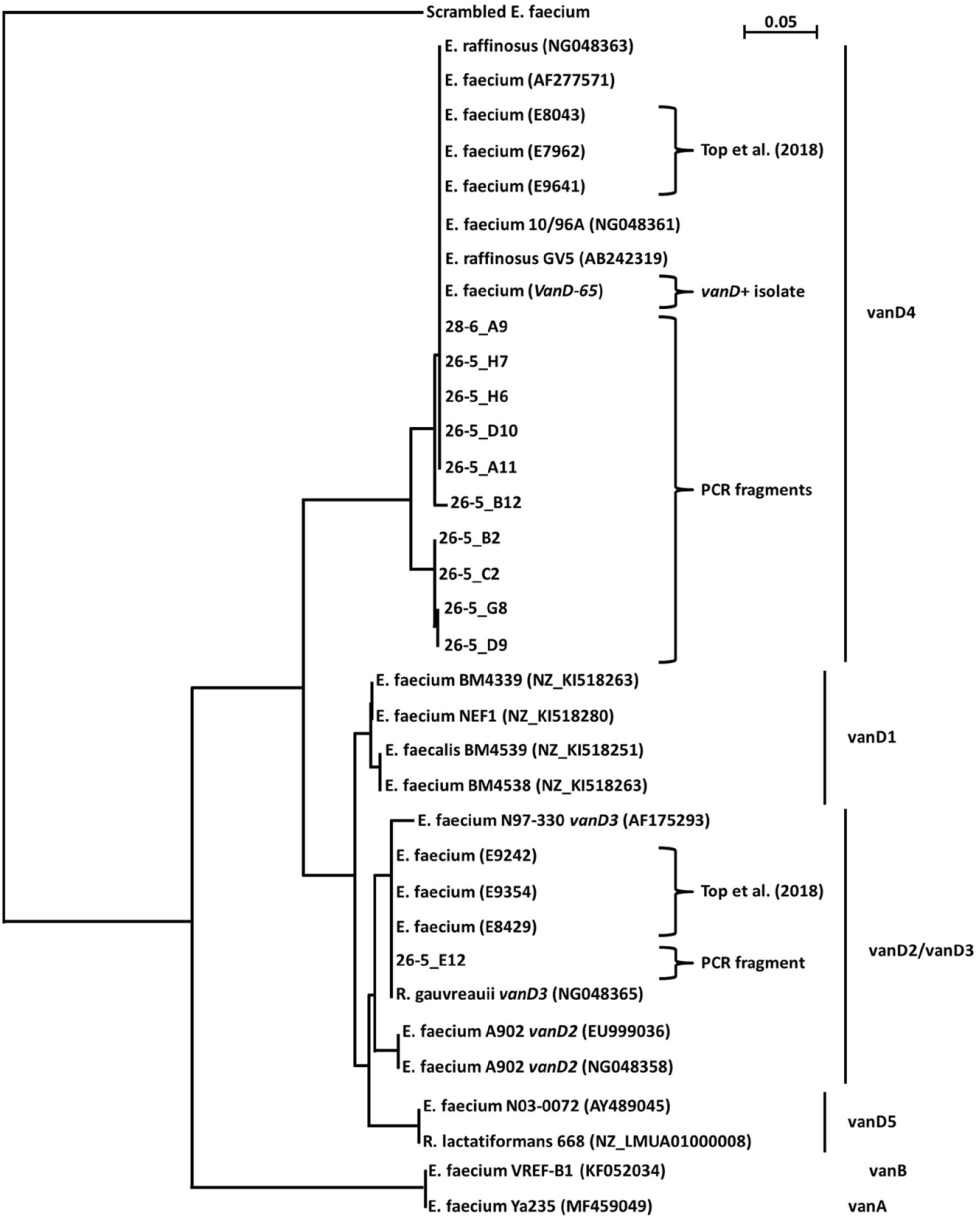
Phylogenetic neighbour-joining (NJ) tree of the *vanD*-positive *E*. *faecium* described in this report and the sequenced *vanD*-PCR fragments (~150 nt). NJ tree was generated and bootstrapped (1000 repetitions) from ClustalX2 multiple alignment of trimmed DNA sequences (Supplement A6). The type of vancomycin-resistance gene is denoted on the right-hand side of the figure, based on the most similar reported *vanD* gene. Further included isolates include the six Dutch *vanD*-positive isolates recently reported^17^ and reference genomes. Accession numbers are given in the figure. The tree was rooted using a randomly scrambled *E*. *faecium* fragment. Bar represents substitutions per site.

Retrospectively, all *vanA*-/*vanB*-negative VRE isolates of the patient (N = 7) were subjected to WGS at the Maastricht University Medical Center (MUMC+). All seven isolates had *vanD*-mediated resistance to vancomycin (Table 1), belonged to ST17/CT154 and whole-genome-MLST showed a maximum of one locus difference between the seven isolates. The *vanD* gene belonged to *vanD4*, as did three out of six of the previous Dutch *vanD*-positive isolates (Fig. 2, Top *et al*.^17^). Comparison of the genetic environment of the *vanD* locus in these four Dutch *vanD*-positive VRE revealed >99.9% homology (Supplement A2A,B). However, our isolate belonged to a different ST/CT and is thus not clonally related to previously described Dutch *vanD*-positive VRE (Table 1, Supplement A2C). The patient also had a vancomycin susceptible *E*. *faecium*, which had been isolated on day 35. Sadly, this isolate could not be retrieved for analysis. Therefore, no clonal relatedness can be inferred between this isolate and the subsequent VRE isolates.

### VRE screening strategy

Upon encountering the *vanD*-positive VRE, the prevalence of *vanD*-positive VRE within our hospital was determined. For this goal, PCRs for *vanC1/2/3* and *vanD* (covering all currently known *vanD1* – *vanD5* sequences) were designed and validated using clinical isolates and transformants (Supplement A3). Next, using these validated PCRs, the prevalence of the *vanA*, *vanB*, *vanC1*, *vanC2/3* and *vanD* genes within our hospital was assessed. In the period of four days, rectal swabs were taken from 360 patients on 12 different wards. In principle, each patient had only one swab taken as part of this monthly point prevalence screening. Each swab was shaken in a TSB broth. Subsequently, the broths were incubated overnight prior to being subjected to PCR analysis for vancomycin-resistance genes (Table 2 below). The prevalence is calculated as the number of PCR-positive broths divided by the total number of broths.

**Table 2:** Prevalence of various vancomycin-resistance genes by PCR in TSB broths inoculated with rectal swabs.

Gene	PCR	
	Positive broths (#)	Prevalence (%)
*vanA*	2	0.6%
*vanB*	38	10.6%
*vanC1*	28	7.8%
*vanC2/3*	26	7.2%
*vanD*	100	27.8%
Total unique broths	139	38.6%

360 rectal swabs were used to inoculate TSB broths. The broths were left to incubate at 37 °C overnight prior to analysis by PCR.

Secondly, to determine whether these genes represented VRE carriage, 47 of the PCR-positive broths were sub-cultured on VRE chromogenic agar to determine VRE carriage (100% of *vanA*-positive (2 out of 2), 100% of *vanB*-positive (38 out of 38), 17.6% of *vanC*-positive (9 out of 51), and 32% of *vanD*-positive (32/100) broths), which resulted in 2 *vanA*-positive *E*. *faecium* (Supplement A5).

Since the VRE-selective agar inhibits growth of *vanC*-containing *E*. *gallinarum* and *E*. *casseliflavus*, another strategy was employed to identify VRE; an additional 16 TSB broths were selected based on their low Ct values for *vanC1*, *vanC2/3*, and *vanD* (i.e. cultures with the highest load); *vanC1* Ct 21–29, *vanC2/3* Ct 17–33, and *vanD* Ct 26–39. These TSB broths were inoculated on both CNA agar and Columbia 5% sheep blood agar, on which a 5 μg vancomycin disc was placed. The rationale to select broths with the lowest Ct values was based on our validation showing that broths with a Ct value over 35.0 for either *vanA* or *vanB* do not result in culturable VRE’s (data not shown).

After 24 h and after 48 h of incubation at 37 °C, colonies that grew within the zone of inhibition were selected and characterized by PCR, MALDI-ToF and antibiotic susceptibility testing (Supplement A5). This resulted in 8 agar plates with growth: 5 vancomycin resistant *vanC1*-positive *E*. *gallinarum* (Ct 21–29, MIC 4 to 16 mg/L), a vancomycin susceptible *vanC2/3*-positive *E*. *casseliflavus* (Ct 17, MIC 1 mg/L) and a vancomycine susceptible *Enterococcus faecalis* negative for *vanA/B/C/D* (MIC 2 mg/L). Additionally, a vancomycin-susceptible *Staphylococcus haemolyticus* was isolated. No *vanD*-positive enterococci were identified.

Given the multitude of broths positive for *vanD* by PCR (100 out of 360; 27.8%), eleven samples were randomly selected among the high-positive PCRs (i.e. low Ct value), sequenced to confirm the specificity of amplification, and aligned with known *vanD*-sequences (Fig. 2 below, Supplement A6). Alignment and BLAST analysis revealed high similarity to the *vanD* sequence of our isolate and to other *vanD* sequences previously found in *E*. *faecium* and *E*. *raffinosus*. One sequence (26-5_E12) is most similar to a *vanD* sequence found in *Ruminococcus gauvreauii*, an anaerobic species.

Lastly, the results of this screening were combined with existing data, enabling calculation of the positive predictive value (PPV) of our *vanA*, *vanB*, and *vanD* PCRs for VRE in a non-outbreak situation in our hospital. Here, the positive predictive value of the PCR indicates which fraction of PCR-positive broths yield VRE when cultured on VRE-selective agar. This resulted in; *vanA* 82% PPV (N = 65), *vanB* 8% PPV (N = 426), *vanD* 0% PPV (N = 100). The PPV of *vanC1/2/3* PCR was not calculated given the low clinical relevance of the microorganisms typically harbouring these resistance genes, and the fact that these microorganisms do not grow on the VRE-selective agars.

## Discussion

In response to the isolation of a *vanD*-positive VRE, PCRs for *vanC1/2/3* and *vanD* were devised and validated to determine the prevalence of *vanC/D*-positive enterococci within our hospital. A hospital-wide screening, comprising 360 patients, did not show any relevant *vanC-* or *vanD-*positive enterococci. The observed prevalence of the *vanB* gene (10.6%) and the *vanD* gene (27.8%) in our hospital are similar to those in literature^13^, whereas the prevalence of the *vanA* gene is lower (0.6% versus 9.3%). For the *vanC* genes, the prevalence of 7.2% (*vanC1*) and 7.8% (*vanC*2/3) in our hospital is higher than in literature^10,20^, as will be discussed below. Culture of *vanC*-positive broths yielded 5 *vanC1*-positive and 1 *vanC2/3*-positive enterococci (success rate 6/14: ~43%). Yet, these *vanC*-positive enterococci have sub-clinical vancomycin MIC values (range: 1–16 mg/L), values which are well achievable in serum^21^.

Previous studies into the prevalence of vancomycin-resistant enterococci used 6 mg/L vancomycin during the enrichment step and this likely has affected the observed prevalence of *vanC*. For example, in Hungary, clinical samples were first grown on VRE selection agars, resulting in a prevalence of 0.19% and 0.3% for *vanC1* and *vanC2*, respectively, among all enterococci-positive samples^10^. In Nigeria, rectal swabs were inoculated in broth containing 6 mg/L vancomycin prior to screening, resulting in a prevalence of 2.8% for *vanC1-* and 0.3% for *vanC2-*positive enterococci^20^. We used broths without any vancomycin and noted a prevalence of 7.8% and 7.2% for *vanC1* and *vanC2/3*, respectively (Table 2). The VRE-selective agar used in this study contains 8 mg/L vancomycin, besides other chemicals that inhibit growth of *vanC*-positive *E*. *gallinarum* and *E*. *casseliflavus* (data not shown). When CNA agar with a disk of vancomycin was used for culture, *vanC*-positive enterococci were isolated in 43% of the tested PCR-positive broths indicating that these enterococci could readily be isolated in case of PCR-positivity. Furthermore, given the relatively low MIC values conferred by *vanC* genes, the majority of *vanC*-containing enterococci possibly will be missed if selection is applied prior to screening. Yet, this is currently not seen as a flaw given the low clinical relevance of *vanC*-positive *E*. *gallinarum*, *E*. *casseliflavus*/*E*. *flavescens*^8–11^.

In contrast, the prevalence of *vanD* apparently is less affected by the use of selective broths since we found a prevalence of 27.8% (the Netherlands) without selective broth and others found 43.8% (Montreal, Canada) and 26.7% (Boston, USA) after enrichment in selective broth containing 6 mg/L vancomycin and 60 mg/L aztreonam^13^. These prevalences are quite similar despite the geographic distance and the presence or absence of selection. Despite the high prevalence, and the fact that the sequenced *vanD* amplicons were most similar to *vanD* genes associated with enterococci (Fig. 2), neither we nor other authors^13^ were able to grow *vanD*-positive enterococci. The fact that the attempts to culture *vanD*-positive enterococci were futile suggests that the *vanD* gene is more present in our commensal microbiota than previously appraised. Furthermore, the fact that the isolated VRE had chromosomally integrated *vanD* suggests that the gene is not readily transferable between bacteria. Hence, where did this *vanD*-positive VRE originate?

One hypothesis is that the patient became colonized within the hospital. However, as described above, we could not isolate further *vanD*-positive VRE from other patients despite multiple attempts and approaches. Moreover, our isolated *vanD*-positive VRE was studied by WGS, revealing it to be an MLST ST17/CT154, a rare type that is currently predominantly encountered in Germany^22^. However, these ST17/CT154 VRE are *vanA*-positive and have different antibiotic gene repertoires suggesting that there is no direct connection between the German isolates and our isolate (Supplement A1).

Thus, it is more likely that the patient had the *vanD* gene present in his gut, either already in an *E*. *faecium* or in other microorganisms. In case of the first, the initial load of VRE was perhaps too low for efficient culture during the first 1.5 month of hospitalization. Vancomycin-based therapy then enabled the VRE to expand while the other fecal microbiota diminished^23,24^. The alternative hypothesis is that the *E*. *faecium* of the patient had acquired the *vanD* gene cluster from neighbouring anaerobic microorganisms, e.g. *Clostridium* spp, *Blautia* spp, or *Ruminococcus* spp^13,25^. Since anaerobic microorganisms are not expected to grow under aerobic conditions, i.e. under our screening protocol, the presence of the vancomycin resistance genes could be detected by PCR in a growth-independent manner while the subsequent screening on agar would not produce any growth. In line with the possibility of *vanD* gene cluster transferal from neighbouring anaerobic microorganisms, the *vanD* gene cluster in our VRE isolate seems to have integrated along with fragments of *Blautia* spp.-related genomic DNA.

Yet, given the high homology of the *vanD* gene cluster in our isolate and three other, older, Dutch *vanD*-positive VRE^17^, this *vanD* gene cluster may once have crossed from *Blautia* spp. into an enterococ and thereafter have spread horizontally to other ST of *E*. *faecium* (Supplement A2A–C). Furthermore, the high homology in these Dutch VRE suggests that our patient acquired a VRE that was already *vanD*-positive. More importantly, if the *vanD* gene cluster is indeed derived from *Blautia* spp., this implies that new resistance mechanisms can be introduced from gut microbiota into enterococci, especially after prolonged treatment with antibiotics (this report)^16^.

Since many clinical laboratories focus on pre-screening for the presence of *vanA* and *vnB* genes by PCR, this could lead to underestimation of *non-vanA/non-vanB* VRE. Therefore, in response to this *vanD*-positive VRE, a PCR for *vanC1/2/3* and *vanD* was designed and validated to determine the prevalence of *vanC/D*-positive enterococci within our hospital. Yet, the screening did not yield any relevant *vanC-* or *vanD-*positive enterococci.

We conclude that the occurrence of a *vanD*-positive VRE is currently still very rare in the clinical setting. Yet, this report and others^16,17^ indicate that *vanD*-positive VRE certainly can be found in patients and, being a VRE, pose a risk for nosocomial spread. Given the high prevalence of *vanD* in rectal swabs within our hospitalized population, and the low PPV of *vanD*-positive PCR, it would not be cost-effective to include the *vanD* PCR in our VRE screening program. However, having validated these *vanC1/2/3* and *vanD* PCRs in our hospital, future *vanA/B*-negative VREs can now be rapidly checked for the presence of a *vanC* and *vanD* gene.

## Materials and Methods

### Surveillance

As part of the routine screening for the presence of vancomycin-resistant *e*nterococci (VRE) within the MUMC+, 360 patients from 12 different wards were screened by taking an rectal swab, which were subsequently brought to the department of Medical Microbiology for analysis. Here, the swabs were suspended in Tryptic soy broth (TSB) medium (Tritium microbiologie, the Netherlands) and then cultured overnight at 37 °C prior to DNA extraction and analysis.

### DNA extraction

200 μL of overnight cultures was extracted on a MagNA Pure 96 system using MagNA Pure LC 96 DNA and viral NA small volume kit (Roche Diagnostics, the Netherlands). Total nucleic acids were eluted in 100 μL eluate.

### Molecular detection of vancomycin-resistance genes

Primers and probes sequences to detect *vanA* and *vanB* genes were obtained from Erik van Hannen (St Antonius Hospital, Nieuwegein, the Netherlands). Primers and probes to detect *vanC* and *vanD* genes were designed in-house for this study. All available sequences of *vanC* and *vanD* genes were obtained from NCBI’s GenBank (Supplement A3), after which all unique sequences were aligned using Clustal Omega. Next, using IDT’s PrimerQuest and OligoAnalyzer tools, primers and a TaqMan probe were designed to match all allele variants of the *vanC1*, *vanC2/3* and *vanD* genes. The primers and probes were analyzed *in-silico* to avoid cross-annealing.

Absence of cross-annealing was validated *in-vitro* against strains harboring either *vanA*, *vanB*, *vanC1*, *vanC2/3* or *vanD* (Supplement A4). The specificity of the PCRs was further validated by testing against; I) reference positive and negative strains and plasmids, II) known negative clinical isolates, and by sequencing PCR products of *vanC1/2/3*- and *vanD*-positive PCRs (Fig. 2). All primer/probe sequences are displayed in Table 3 below.

**Table 3:** List of primers and probes used in this study.

mCMVgb_forward		AGGGCTTGGAGAGGACCTACA	
mCMVgb_reverse		GCCCGTCGGCAGTCTAGTC	
mCMVgb_probe	NED	AGCTAGACGACAGCCAACGCAACGA	BHQ2
vanA_forward		GCCGGAAAAAGGCTCTGAA	
vanA_reverse		TCCTCGCTCCTCTGCTGAA	
vanA_probe	FAM	ACGCAGTTATAACCGTTCCCGCAGACC	BHQ1
vanB_forward		CGCAGCTTGCATGGACAA	
vanR_reverse		GGCGATGCCCGCATT	
vanB_probe	VIC	TCACTGGCCTACATTC	MGB-NFQ
vanC1_forward		CTTATGTTGGTTGCCATGTCG	
vanC1_reverse		CGATTGTGGCAGGATCGTT	
vanC1_probe	FAM	TGGCTCTTGCATCAACTTGCTGATACCA	BHQ1
vanC2/3_forward		GCACTCCAATCATCTCCCTATG	
vanC2/3_reverse		CAYGTGTCTTGTCGGATGTT	
vanC2/3_probe	JOE	TAYGACCTCTCTTTGATCGGGATCRCC	BHQ1
vanD_forward		GCCATACTGGGAAAYGRAAA	
vanD_reverse		CAGCCAAGTAYCCGGTAAATC	
vanD_probe	FAM	TCCGGCTGTGCTTCCTGATGRATC	BHQ1

### Controls and template plasmids

A plasmid containing murine CMV glycoprotein B (*mCMV-gb*) was used as internal spike to check for efficient DNA extraction and PCR-inhibitory compounds. Control plasmids for *vanC1* and van*C2/3* were constructed by cloning the corresponding PCR amplicon into a pGEM-T easy vector (Promega Corporation, Madison, WI, USA). The constructs were transformed into *E*. *coli* TOP10 and used as positive control for DNA extraction and PCR detection. Clinical isolates of enterococci containing *vanA*, *vanB* and *vanD* were used as positive controls.

### Amplification and detection

PCR of *vanA*, *vanB* and the internal control *mCMVgb* was performed in a multiplex reaction. Similarly *vanC1* and *vanC2/3* were combined in a multiplex reaction, whereas *vanD* was a simplex reaction. Primer-probe concentrations for *vanA* and *vanB* were 800 nM of each primer and 200 nM probe. The PCR of *mCMVgb*, *vanC1*, *vanC2/3* and *vanD* contained 300 nM of each primer and 200 nM probe. Each PCR was performed in a total volume of 25 μL consisting of 12.5 μL ABsolute QPCR ROX Mix (Thermo Scientific, Waltham, MA, USA), the primer/probe mixture at the concentrations given above and 5 μL of DNA eluate. Amplification was performed on a 7900HT Fast Real-Time PCR System (Applied Biosystems, Foster City, CA, USA) under the following conditions: 15 minutes at 95 °C, followed by 42 cycles of 15 seconds at 95 °C and 1 minute at 60 °C.

### Isolation and culture of vancomycin-resistant microorganisms

Based on the PCR screening, all TSB cultures that were positive for *vanA* or *vanB* were inoculated onto VRE-selective chromogenic agar (bioMérieux, Marcy-l′Etoile, France). In addition, a selection of the *vanC* and *vanD* PCR-positive TSB cultures were streaked on VRE-selective agar to check for presence of VRE. TSB cultures positive for *vanC* or *vanD* were streaked onto CNA agar (colistin, nalidixic acid) and blood agar (both from Bection-Dickinson, USA) on which a 5 μg vancomycin disc (Rosco diagnostica, Danmark) was placed. All agar plates were incubated for at least 48 h and checked after 24 h and 48 h of incubation for growth. Colonies within the inhibition zone were further cultured and characterized by screening axenic microbial cultures by PCR for the presence of *vanC1*, *vanC2/3*, or *vanD*. Isolates positive for a vancomycin-resistance gene were identified using VITEK MS (MALDI-TOF, bioMérieux). Antibiotic resistance was determined using VITEK II and E-test (bioMérieux), according to manufacturer’ instructions. As controls, agar plates were inoculated with a 0.5mcFarland suspension of fresh grown bacterial isolates. These controls were prepared by mixing a VSE with a VRE in the ratio of 95:5 volume/volume %, respectively. For *E*. *faecalis*, this was ATCC 29212 (VSE) and ATCC 51299 (*vanB*-positive VRE with low-level resistance). For *E*. *faecium*, clinical isolates were used (one each for *vanA*, *vanB* and *vanD*).

### Whole genome sequencing

Genomic DNA was purified using NucliSENS easyMAG (bioMérieux) following the manufacturer’s instructions, and libraries were prepared using a Nextera XT Kit (Illumina, Little Chesterford, UK). Illumina MiSeq 250 bp paired end sequencing was applied. Genome assembly of *E*. *faecium* was performed with SPAdes genome assembler, version 3.10.0^26^. The assembled genome was submitted to ResFinder 2.1 (http://cge.cbs.dtu.dk/services/ResFinder/), using default settings (90% sequence similarity and ≥60% coverage length) which identifies acquired antimicrobial resistance genes^19^.

### cgMLST and wgMLST analysis

cgMLST was performed using the official E. faecium cgMLST scheme provided by Ridom SeqSphere+ (v5.1.0). Similar ST17/CT154 genomes were identified at cgMLST.org and downloaded as draft genomes. wgMLST was performed using the BioNumerics (v7.6.3) WGS plugin with wgMLST *E*. *faecium* scheme.

### Amplicon sequencing

Sequencing of PCR products was performed as described before^27^ using the PCR primers and an ABI BigDye Terminator v1.1 cycle sequencing kit (Applied Biosystems, Foster City, CA, USA). Sequencing data were obtained by using an ABI 3730 DNA Analyzer (Applied Biosystems) and were analyzed using BLAST (http://blast.ncbi.nlm.nih.gov/Blast.cgi). 16S sequencing was performed as previously described^28^.

### Mauve analysis

Mauve v20150222 build 10 was used to compare the DNA contig carrying *vanD* gene cluster in VRE to the most similar contigs carrying *vanD* gene available in Genbank and published by Top *et al*.^17^. DNA similarity between these *vanD*-carrying contigs was analyzed by performing pairwise BLASTn analysis.

### Phylogenetic analysis of contigs carrying the *vanD* gene clusters

The same DNA contigs from the MAUVE analysis were also subjected to phylogenetic analysis based on variations in single Nucleotide Polymorphisms (SNPs) to determine their genetic relatedness. The 192 kb contig 1 from strain *vanD-*65 was used as reference and the phylogenetic distance between the selected contigs were calculated using CSIPhylogeny (https://cge.cbs.dtu.dk/services/CSIPhylogeny/) with a pruning distance of 100 bp.

### Ethics statement

All materials were derived from routine clinical diagnostics, and were de-identified prior to analysis. Neither information nor samples have been collected specifically for this study. All data were analyzed anonymously.

## Supplementary information

Supplementary info

## Data Availability

The Whole Genome Shotgun project has been deposited at DDBJ/ENA/GenBank under bioproject PRJDB7459, Biosamples: SAMD00141057 – SAMD00141063.
