# Supplementary material for: Appearance of vanD-positive Enterococcus faecium in a tertiary hospital in the Netherlands: prevalence of vanC and vanD in hospitalized patients
Source: Sci Rep. 2019 May 6;9:6949. doi: 10.1038/s41598-019-42824-4 (PMC6502811; doi:10.1038/s41598-019-42824-4)
Supplement: Supplementary file 1 — Supplementary info [file 41598_2019_42824_MOESM1_ESM.pdf]

Appearance of *vanD*-positive *Enterococcus faecium* in a tertiary hospital in the Netherlands: prevalence of *vanC* and *vanD* in hospitalized patients

Jacky Flipse[1,2*], Christian J. von Wintersdorff[1], Julius M. van Niekerk[1], Casper Jamin[1], Frank H. van Tiel[1], Henrik Hasman[3], Lieke B. van Alphen[1*]

Affillations:

1.Department of Medical Microbiology, Care and Public Health Research Institute (CAPHRI), Maastricht University Medical Center+, Maastricht, The Netherlands

2. present address: Laboratory for Medical Microbiology and Infectious Diseases, Isala, Zwolle, the Netherlands

3. Department of Bacteria, Parasites and Fungi, Statens Serum Institut, Copenhagen, Denmark

* correspondence could be directed to: j.c.flipse@isala.nl or lieke.van.alphen@mumc.nl

Supplement A1: resistance genes present in VRE belonging to MLST17, CT154

Genome ID:	this study	RID057382	RID039541	RID025089	EOO3841	EOO4012	EOO6405
Aminoglycoside-resistance	ant(6)-Ia	ant(6)-Ia			ant(6)-Ia	ant(6)-Ia	ant(6)-Ia
	aph(3')-III					spc	
Glycopeptide-resistance	VanR-D	VanZ-A	VanX-A	VanZ-A	VanX-A	VanR-A	VanR-A
	VanS-D	VanR-A	VanH-A	VanR-A	VanH-A	VanH-A	VanH-A
	VanY-D	VanH-A	VanS-A	VanH-A	VanS-A	VanA	VanA
	VanH-D	VanA	VanR-A	VanA	VanR-A	VanX-A	VanX-A
	VanD	VanY-A	VanZ-A	VanY-A	VanZ-A	VanZ-A	VanZ-A
	VanX-D	VanX-A	VanY-A	VanX-A	VanY-A	VanY-A	VanY-A
		VanS-A	VanA	VanS-A	VanA	VanS-A	VanS-A
MLS-resistance	erm(B)	erm(B)	erm(B)	erm(B)	erm(B)	erm(B)	erm(B)
	msr(C)	msr(C)	msr(C)	msr(C)	msr(C)	msr(C)	msr(C)
						erm(A)	
Phenicol-resistance		cat(pC221)	cat(pC221)		cat(pC221)	cat(pC221)	cat(pC221)
Tetracycline-resistance					tet(U)	tet(U)	

Table A1: overview of resistance genes present in VRE belonging to MLST17, CT154. MLS: Macrolide, Lincosamide and Streptogramin B

Supplement A2: MLST minimum-spanning tree

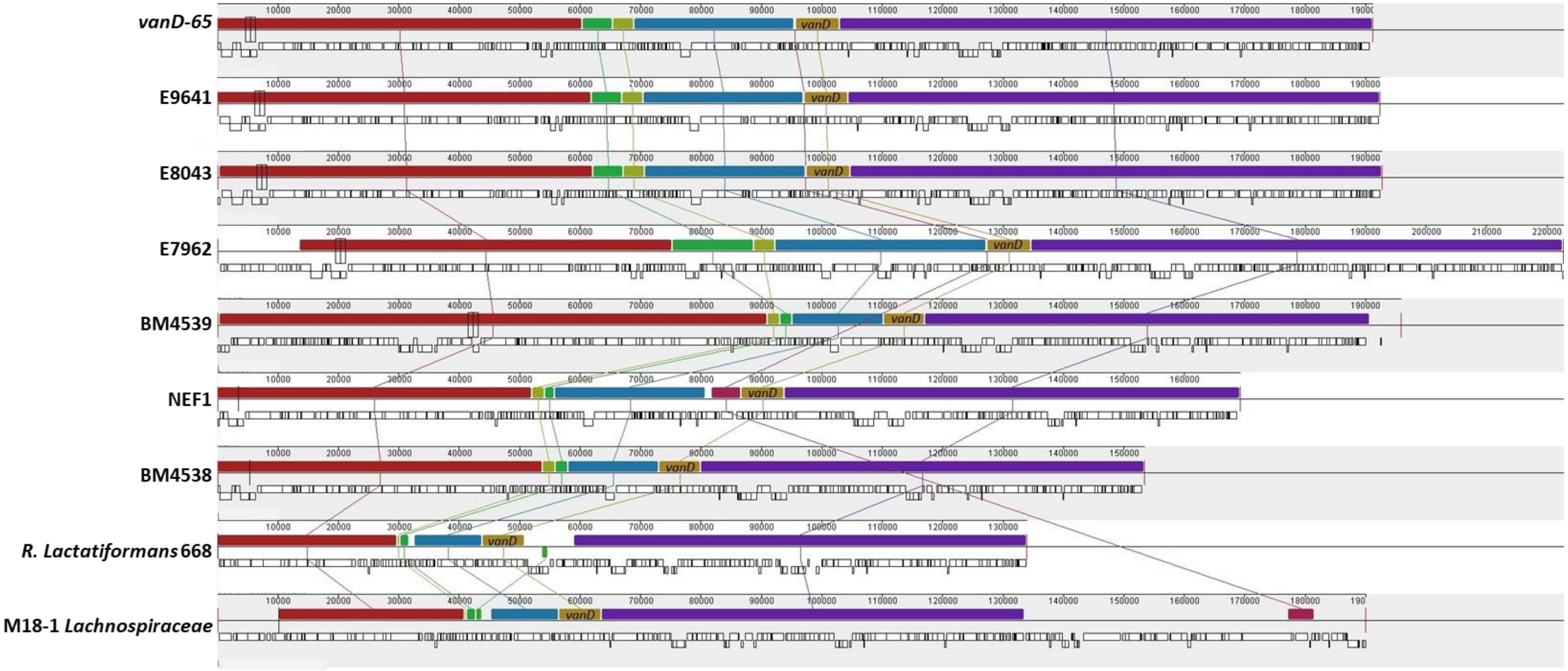

Figure A2A: Multiple alignment analysis using MAUVE (v20150226 build 10) of the genomic environments of the *vanD*-cluster in *vanD*-positive VRE and other microorganisms based on the *vanD*-positive contigs of our isolate (top, isolate of the 65th day of hospitalization), compared with three *vanD4*-positive isolates (Top et al. [1]; E9641, E8043, E7962) and 5 other reference genomes: *E. faecium* (NEF1, BM4538), *E. faecalis* (BM4539), *R. lactatiformans* 668, Lachnospiraceae bacterium M18-1. Regions of high homology are denoted by coloring and vertical lines. The positions of the *vanD* gene clusters are indicated with brown color and marked “*vanD*”

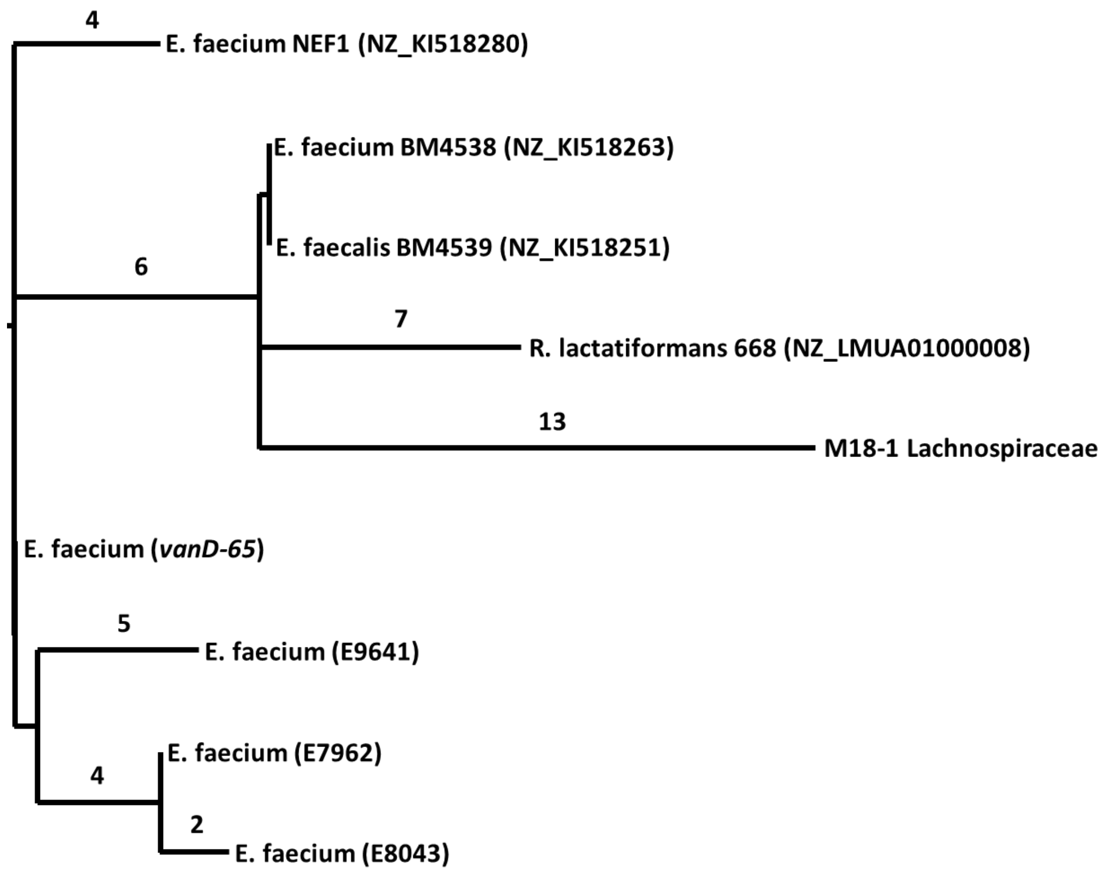

Figure A2B: Phylogenetic comparison of the genomic enviroments of the *vanD*-positive VRE and other microorganisms using CSIPhylogeny (v1.4). The *vanD*-positive contig 1 of *vanD-65* served as a reference to calculate the SNP distance between this isolate and other Dutch *vanD4*-positive VRE's (Top et al. [1]; E9641, E8043, E7962) as well as 5 other reference genomes: *E. faecium* (NEF1, BM4538), *E. faecalis* (BM4539), *R. lactatiformans* 668, Lachnospiraceae bacterium M18-1. SNP distances are indicated above the horizontal lines.

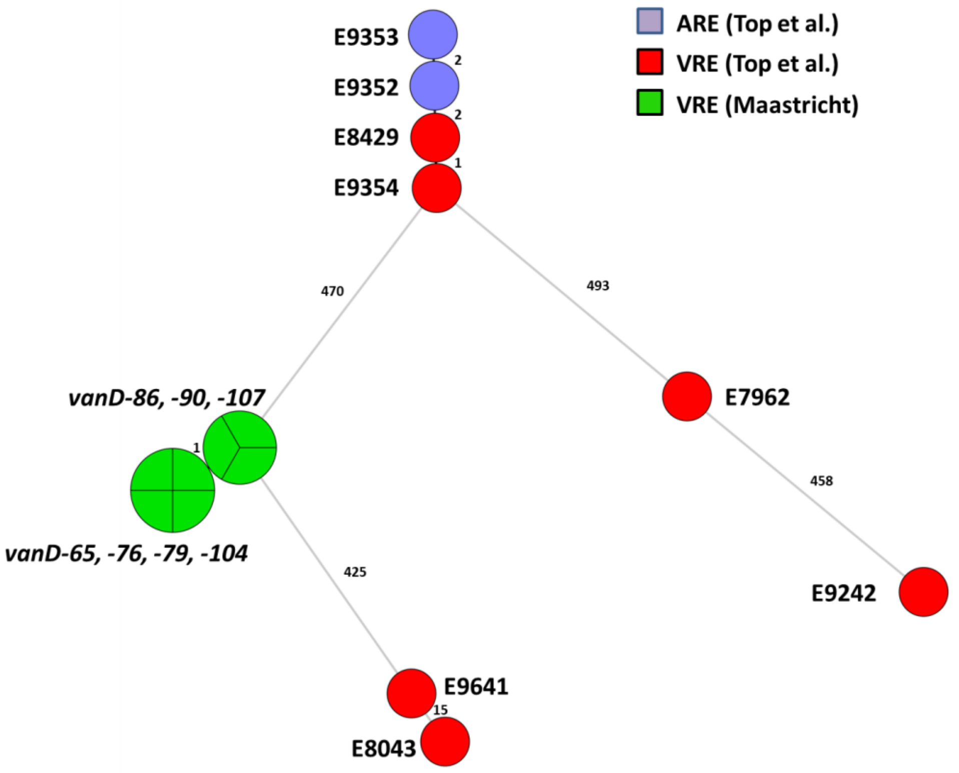

Figure A2C: minimum spanning tree comparing the seven *vanD*-positive *E. faecium* isolates of our casus (green) with eight Dutch *E. faecium* isolates previously described by Top et al., which are either *vanD*-positive (VRE, red) or ampicillin resistant (ARE, purple). Numbers reflect distance based on wgMLST.

Reference:

1. Top, J. *et al.* Identification of a novel genomic island associated with vanD-type vancomycin-resistance in six Dutch vancomycin-resistant Enterococcus faecium. *Antimicrob Agents Chemother*, doi:10.1128/AAC.01793-17 (2018).

Supplement A3: Sequences of vanC1/2/3 and vanD genes used to design primers and probes used in this study.

Gene	Gen bank ID	Gene	Gen bank ID	Gene	Gen bank ID
vanC1		*vanC2/3*		*vanD*	
	AY870598.1		AY033764.1		Isolate; *vanD-65*
	AY870595.1		EU151759.1		AB183866.1_NG048363.1
	EU151769.1		EU151755.1		AF277571.1_NG048361.1
	AF162694.1		CP004856.1		AY489045.1_NG048362.1
	JX435778.1		EU151757.1		AF153050.1_NG048358.1
	DQ022190.1		EU151761.1		EF508033.1_NG048365.1
	JX220984.1		EU151758.1		AF130997.1_NG048359.1
	AY786180.1		EU151754.1		AF175293.1_NG048360.1
	EU151772.1		EU151760.1		
	EU151768.1		EU151762.1		
	EU151770.1		CP014067.1		
	EU151767.1		EU151753.1		
	JN003409.1		EU151752.1		
	KU296945.1		EU151756.1		
			EU151763.1		

Supplement A3: Gene sequences used to design primers and probe against *vanC1*, *vanC2/3*, and *vanD*. Known *vanC1*, *vanC2/3*, and *vanD* genes were downloaded from the NCBI genbank by searching against a gene sequence (e.g. *vanC1*: NG048343.1, *vanC2/3*: AY033764.1, and *vanD*: NG048362.1). Hits have been downloaded and duplicates removed prior to aligning unique sequences with Clustal Omega. Next, using IDT's PrimerQuest and OligoAnalyzer tools, primers and a TaqMan probe were designed to match all allele variants of the *vanC1*, *vanC2/3* and *vanD* genes.

Supplement A4: validation of vanA, vanB, vanC1, vanC2/3, vanD PCRs

Validation *vanA/B/C/D*

Copies per PCR	VRE *vanA* (Ct)	VRE *vanB* (Ct)
10^3	25,33	27,99
10^2	28,63	31,80
50	29,71	32,39
20	31,00	34,14
10	31,63	35,64
5	33,10	36,29
2	34,88	Undet.
1 *	34,37 / Undet.	Undet. / Undet.
0 *	Undet. / Undet.	Undet. / Undet.

Table A4.1: PCR results obtained in dilution series of Amplirun® *Enterococcus faecium* (*vanA*) DNA and Amplirun® *Enterococcus faecium* (*vanB*) DNA. * test is performed in duplicate. Undet.: undetectable

Copies per PCR	VRE *vanC1* (Ct)	VRE *vanC2/3* (Ct)	VRE *vanD* (Ct)
$2{\cdot}10^5$	12,94	12,90	15,44
$2{\cdot}10^4$	16,50	16,40	18,98
$2{\cdot}10^3$	19,96	19,79	22,28
$2{\cdot}10^2$	23,34	23,09	25,93
$2{\cdot}10^1$	26,74	26,40	29,24
$2{\cdot}10^0$	N.D.	N.D.	32,72

Table A4.2: PCR results obtained in dilution series of *in house* standard curve. * test is performed in duplicate. Undet.: undetectable. N.D.: Not determined

Having established the linearity of the PCR, we further validated the *vanA/B* PCRs against 400 routine rectal cultures. Fecal swabs were processed as described in the Materials and Methods and were subjected to both *vanA/B* PCR and culture. Of these, 51 were culture and PCR positive, 2 were PCR positive of patients who were VRE-positive in other fecal swabs. Two samples were PCR negative, yet VRE-positive by culture. 297 samples were negative in both culture and PCR.

Additionally, the *vanA/B* PCR was further validated against an external quality panel consisting of 4 *vanA*-positive *E. faecium*, 5 *vanB*-positive enterococci (*E. faecalis* 3x, *E. faecium* 1x, *E. gallinarum* 1x). The *vanC1*, *vanC2/3*, *vanD* PCRs were validated against clinical isolates of enterococci positive for *vanC* (8x) and *vanD* (1x), as well as *E. coli* harbouring a plasmid containing the *vanC1*, *vanC2/3*, or *vanD* insertion. All were positively identified by the respective PCR's.

None of the five vancomycin-resistance PCR's cross-reacted with non-VRE enterococci (4x), or with clinical isolates of faecal microbiota e.g. *E. coli* (3x), *P. mirabilis* (1x), *S. marcescens* (1x), *P. aeruginosa* (1x). Moreover, no cross-reactivity was seen between specific PCRs with discordant targets as shown by: *vanA*-positive enterococci (6x), *vanB*-positive enterococci (6x), *vanC*-positive enterococci (12x), *vanD*-positive enterococci (1x).

Supplement A5: Culture results of selected PCR-positive broths

Gene	Broths selected for culture	Culture		VRE-positive
		VRE agar	CNA agar	
vanA	2	2	0	2
vanB	38	38	1*	0
vanC1	15	6	9	5
vanC2/3	8	3	5	0
vanD	43	32	12*	0
Total unique broths	63	47	16	7

Table A5: Overview of results derived from broths inoculated with rectal swabs. Rectal swabs were used to inoculate TSB broths. The broths then incubated at 37°C overnight prior to analysis by PCR. 38.6% of the broths were positive for one or multiple *van* genes by PCR. Of the PCR-positive broths, all *vanA*- and *vanB*-positive broths were plated on VRE-selective agar. In case of broths positive for *vanC* or *vanD* , several were plated on VRE-selective agar due to presence of either *vanA* or *vanB*. An additional 16 broths were selected based on their low Ct value. These broths were inoculated on CNA agar with a disc of vancomycin, as an alternative approach to find vancomycin-resistant enterococci. VRE are defined as an *Enterococcus* with a MIC value of >4 mg/L for vancomycin[1]. * 1 broth was *vanB*+ *vanD*+, and tested on both VRE selective agar and CNA agar.

Reference:

1. EUCAST. Breakpoint tables for interpretation of MICs and zone diameters. Version 8.0, 2018. (2018).

Supplement A6: full-length *vanD*-PCR-sequences obtained in this study

>26-5_A11_D
ACCGGCGAGGTCGATCAGATTGAATTGAAACACGGGTTTTTTAAGATCCATCAGGAAGCA
CAGCCGGAAAAGGGGTCTGAAAATGCTGTGATTAGAGTTCCAGCCGCCCTGCCGGATGAAGTTA
GGGAGCAGATTCAGGAAACGGCGAA

>26-5_B2_D
TCGCCGGTGAGGTCGATCAGATTGAATTGAAACACGGGTTTTTTAAAATCCATCAGGAAG
CACAGCCGGAAAAGGGGTCTGAAAATGCTGTGATCAGAGTTCCGGCCGCCCTGCCGGATGAAGT
AAGGGAGCAGATTCAGGAA

>26-5_B12_D
CGCCGGCGAGGTCGATCAGATTGAATTGAAACACGGGTTTTTTAAGATCCATCAGGAAGC
ACAGCCGGAAAAGGGGTCTGAAAATGCTGTGATTAGAGTTCCAGCCGCCCTGCCAGATGAAGTT
AGGGAGCAGATTCAGGAA

>26-5_C2_D
CGCCGGTGAGGTCGATCAGATTGAATTGAAACACGGGTTTTTTAAAATCCATCAGGAAGC
ACAGCCGGAAAAGGGGTCTGAAAATGCTGTGATCAGAGTTCCGGCCGCCCTGCCGGATGAAGTA
AGGGAGCAGATTCAGGAA

>26-5_D9_D
CAGCCAAGTATCCGGTAAATCTTCTTCGCCGTTTCCTGAATCTGCTCCCTTACTTCATCCG
GCAGGGCGGCCGGAACTCCTGATCACAGCATTTTCAGACCCCTTTTCCGGCTGTGCTTCCTGAT
GGATTTTAAAAAACCCGTGTTTCAATTCATAC

>26-5_D10_D
ACCGGCGAGGTCGATCAGATTGAATTGAAACACGGGTTTTTTAAGATCCATCAGGAAGCA
CAGCCGGAAAAGGGGTCTGAAAATGCTGTGATTAGAGTTCCAGCCGCCCTGCCGGATGAAGTTA
GGGAGCAGATTCAGGAA

>26-5_E12_D
GCTTGCGAGGTGGATCAGATTGAGCTGAGACACGGCTTTTTTAAGATTCATCAGGAAGCA
CAGCCGGAGAAGGGATCTGAAAATGCAGTCATCCGAGTTCCAGCCGCCTTACCGGATGAGGTAA
GAGAACGGATTCAGAAAACA

>26-5_G8_D
CGGGCTTTTTTAAAATCCATCAGGAAGCACAGCCGGAAAAGGGGTCTGAAAATGCATGTG
ATCAGAGTTCCGGCCGCCCTGCCGGATGAAGTAAGGGAGCAGATTCAGGAA

>26-5_H6_D
CGGCGAGGTCGATCAGATTGAATTGAAACACGGGTTTTTTAAGATCCATCAGGAAGCACA
GCCGGAAAAGGGGTCTGAAAATGCTGTGATTAGAGTTCCAGCCGCCCTGCCGGATGAAGTTAGG
GAGCAGATTCAGGAA

>26-5_H7_D
ACCGGCGAGGTCGATCAGAATGAATTGAAACACGGGTTTTTTAAGATCCATCAGGAAGCA
CAGCCGGAAAAGGGGTCTGAAAATGCTGTGATTAGAGTTCCAGCCGCCCTGCCGGATGAAGTTA
GGGAGCAGATTCAGGAA

>28-6_A9_D
CGGCGAGGTCGATCAGATTGAATTGAAACACGGGTTTTTTAAGATCCATCAGGAAGCACA
GCCGGAAAAGGGGTCTGAAAATGCTGTGATTAGAGTTCCAGCCGCCCTGCCGGATGAAGTTAGG
GAGCAGATTCAGGAAACGGCGAAGA
